# Marshall Hall’s Ready Method in Asphyxia of the Newly-Born, Etc.; Hysteria from Chloroform

**Published:** 1858-05

**Authors:** J. W. Benson


ARTICLE IV.

READ BEFORE THE COOK CO. MEDICAL SOCIETY, BY J. W. BENSON, M.D.

### MARSHALL HALL'S READY METHOD IN ASPHYXIA OF THE NEWLY-BORN, ETC.

Gentlemen,—Since our last meeting, I have had occasion to test and profit by the efficacy of Marshall Hall's ready method in asphyxia of the newly-born.

On the 9th January, my professional services were required in the case of Mrs. H. After a somewhat singular and rapid labor, the head of the child emerged. The umbilical cord was twisted around its neck. Some delay occurred before complete delivery was effected. The child was asphyxiated; no evidence of life was apparent. There was not any discoloration from congestion about the lips or fingers, but the entire surface was of a bronze color. I immediately exposed her face to the air, without removing her from the bed. Placing my right hand expanded over the chest, and my left posteriorly and upon the shoulder, her axilla resting between my thumb and forefinger, I was enabled to place her alternately upon her side and chest, while by the disposition of my hands I could aid the contractions of the chest at proper periods. No other means whatever were resorted to, and after a few minutes (ten at least, according to the bystanders, but rather less in my own opinion), I had the satisfaction of observing efforts at respiration, which although feeble at first, soon became more than sufficient. A too active condition supervened, the lips and fingers became blue, a frothy saliva flowed from the mouth, but by the application of heat and friction to the surface and extremities, these symptoms soon passed away. The result of this case induced a retrospective glance at my experience in similar cases, and I must confess that, so far as appearances warranted, or human nature could make comparisons, I have lost cases, which, by this method, might have been saved.

### HYSTERIA FROM CHLOROFORM.

On the 22d January, I was called to visit Mrs. M. She had inhaled chloroform, the previous evening, because of severe pain in her chest. Her extremities were cold, her pulse small and frequent. She was vomiting incessantly, was perfectly conscious, but speechless.

The pain in her chest recurred in apparently agonizing paroxysms, during which by pantomime she well described the globus hystericus. Her idiosyncrasies from childhood were many and peculiar. She has been insane. I had some of the ammoniated tincture of valerian which I proposed to give her, but the odor of it induced so severe a paroxysm that those around feared she was dying. Determined then to depend more upon a mental impression than medication, I remarked that there need not be the slightest apprehension regarding her case, for I was satisfied that I could arrest the vomiting and allay the pain in less than ten minutes. Dropping some of the compound spirits of lavender into water, I ordered a teaspoonful to be given and repeated in two hours if necessary. The first dose was sufficient. She fell into a quiet slumber, from which she awoke in about two hours. A modified recurrence of the vomiting was arrested by a bowl of oyster soup.

In reference to my observation of the effects of chloroform in different cases, the following may be briefly noted:

Case 1. A lad upon whom I operated for hare lip. In this case there was an almost instantaneous depression of the vital powers. He sank pulseless, but soon revived.

Case 2. That of a negro woman, whose mamma I removed. In this case there was profound stupor for several hours after the completion of the operation.

Case 3. A child, aged 3½ years, upon whom I operated for stone in the bladder. In this case there was high excitation and wild raving delirium, breaking out in the fiercest oaths and imprecations, while at the same time there was not any perceptible consciousness of pain.

Case 4. That of a gentleman, upon whom I operated for stricture by subcutaneous incision. In this case there was perfect consciousness of all that was passing and of every word that was spoken, but complete insensibility to pain. The gentleman, who is a high official, stated that he felt the knife dividing the part, but the sensation was not accompanied by the slightest pain.

CASE 5. A man whose leg I amputated. In this case, well marked opisthotonos was speedily produced, and we were compelled to discontinue its use and resort to stimulants. I have not seen death result from its administration. Whether the different effects I have alluded to, were caused by the different qualities of the article used, or by peculiarities of constitution in the individual cases, I am not prepared to determine. I cannot agree with those who attribute all singular and morbific effects to the deleterious quality of the impure article. I am satisfied that, however pure it may be, there are cases, such as numbers one and five, in which it would soon cause death.

